# Impact of protein intake and nutritional status on the clinical outcome of critically ill patients

**DOI:** 10.5935/0103-507X.20190035

**Published:** 2019

**Authors:** Helânia Virginia Dantas dos Santos, Izabelle Silva de Araújo

**Affiliations:** 1 Hospital Universitário, Universidade Federal do Vale do São Francisco - Petrolina (PE), Brasil.

**Keywords:** Nutritional status, Proteins, Critical illness, Estado nutricional, Proteínas, Estado terminal

## Abstract

**Objective:**

To evaluate the association of nutritional status and protein intake with the clinical outcomes of critically ill patients receiving enteral nutrition therapy in an intensive care unit.

**Methods:**

A retrospective observational analytical study was performed by collecting secondary data recorded in medical records of patients ≥ 18 years of age who were admitted to the intensive care unit and who received exclusive enteral nutrition therapy for at least 72 hours in 2017. Nutritional status was assessed by body mass index and arm circumference. For the estimation of protein requirements, the recommendation of the American Society for Parenteral and Enteral Nutrition was considered. Nutritional adequacy was assessed by the daily collection of prescribed and administered enteral formula. In the analyses, parametric and nonparametric tests were used, and significance was set at p <0.05.

**Results:**

Of the 188 patients evaluated, 71.3% were male. The median age of the patients was 48.5 years (31.0 - 63.75). The main clinical diagnosis was trauma (46.3%), and eutrophic was the most frequent nutritional status (54.8% according to body mass index and 46.4% according to arm circumference). Protein adequacy was not attained in 56.4% of patients, and only 46.8% reached the minimum protein recommendation. The occurrence of mortality was associated with nutritional diagnosis, body mass index (p = 0.023), arm circumference (p = 0.041) and protein adequacy (p = 0.012).

**Conclusion:**

Nutritional status and protein intake were significantly associated with the clinical outcomes of critically ill patients.

## Abstract (pt)

**Objetivo:**

Avaliar a associação do estado nutricional e do consumo proteico com o desfecho clínico de pacientes críticos em uso de terapia nutricional enteral em uma unidade de terapia intensiva.

**Métodos:**

Estudo retrospectivo de caráter observacional analítico, realizado por meio da coleta de dados secundários registrados nos prontuários de pacientes ≥ 18 anos, internados na unidade de terapia intensiva e que receberam terapia nutricional enteral exclusiva por pelo menos 72 horas em 2017. O estado nutricional foi avaliado pelo índice de massa corporal e pela circunferência do braço. Para a estimativa das necessidades proteicas, foi considerada a recomendação da *American Society for Parenteral and Enteral Nutrition*. A adequação nutricional foi realizada por meio da coleta diária do volume da fórmula enteral prescrita e administrada. Nas análises, utilizaram-se testes paramétricos e não paramétricos e foi considerado significante p < 0,05.

**Resultados:**

Dos 188 pacientes avaliados, 71,3% eram do sexo masculino, com idade mediana de 48,5 anos (31,0 - 63,75). O principal diagnóstico clínico foi o trauma (46,3%) e a eutrofia foi o estado nutricional mais frequente (54,8%), segundo o índice de massa corporal, e de 46,4% pela circunferência braquial. A adequação proteica esteve insuficiente em 56,4% dos pacientes e apenas 46,8% atingiram a recomendação proteica mínima. A ocorrência de mortalidade esteve associada ao diagnóstico nutricional, ao índice de massa corporal (p = 0,023) e à circunferência do braço (p = 0,041), assim como a adequação proteica (p = 0,012).

**Conclusão:**

O estado nutricional e o consumo proteico estiveram associados de forma siginificante ao desfecho clínico dos pacientes críticos.

## INTRODUCTION

Critical patients are typically characterized by a state of catabolic stress and a systemic inflammatory response. This inflammatory response is also related to complications that lead to increased infectious morbidity, multiple organ dysfunction, prolonged hospitalization and an increased mortality rate.^(1)^

Patients admitted to intensive care units (ICUs) have a prevalence of malnutrition above 35% at admission. Even when well nourished, patients with trauma tend to develop protein and calorie malnutrition after admission to the hospital. This nutritional condition is also strongly associated with delayed wound healing, increased infectious complications, prolonged hospitalization and increased hospital costs.^(1,2)^

Studies of critically ill patients have found that 40% have weight loss above 10 kg in the period immediately after admission to the ICU. This weight loss may be associated with the increased metabolic rate of these patients and with the impaired use of nutritional substrates. In addition, nutritional needs vary significantly according to the critical condition of the patient, which makes the proper administration of nutritional support to these patients even more difficult.^(3)^

The importance of nutritional support for critically ill ICU patients has been recognized, and several aspects of nutritional care have been investigated in randomized trials over the past few years.^(4)^

The priority in the nutritional therapy of these patients should be protein intake, and enteral formulas should thus be selected according to their protein content to obtain the recommended amount, which is 1.2 to 2g/kg/day.^(5)^ However, some factors may interfere with adequate enteral nutrition, such as late onset of nutritional therapy, frequent surgeries and procedures and the presence of postoperative ileus, among others.^(2)^

The objective of this study was to evaluate the association of nutritional status and protein intake with the clinical outcomes of critically ill patients undergoing enteral nutrition therapy (ENT) only in an ICU of a university hospital.

## METHODS

A retrospective observational study was conducted based on the analytical epidemiological model by means of secondary data recorded in the medical records of adult patients (≥ 18 years old) of both sexes who were admitted to the ICU of a university hospital of the Sertão subregion of Pernambuco state (Brazil), received only ENT for at least 72 hours and were followed-up until ENT weaning or until discharge from the ICU in the period from January to December 2017.

The nutritional status of all patients was evaluated by body mass index (BMI). The adults were classified according to the World Health Organization,^(6)^ and the elderly were classified according to Lipschitz.^(7)^ Weight and height data were obtained from medical records or were provided by the patient. In the absence of this information, weight and height were estimated up to 48 hours after ICU admission by means of anthropometric measures of knee height and arm circumference (AC).

After obtaining the measurements, the data were applied to predictive equations according to sex, ethnicity and age proposed by Chumlea et al.^(8,9)^ AC adequacy was also used to classify the nutritional status according to the Third National Health and Nutrition Examination Survey (NHANES III).^(10)^ Patients with visible signs of edema in the AC region during the nutritional assessment were not assessed.

All patients subjected to ENT received liquid industrialized formulas through an enteral feeding tube in an open system. Enteral nutrition therapy was administered according to specific instructions. Administration was performed intermittently six times a day at three-hour intervals with a nocturnal pause of six hours and was controlled by infusion pumps. Standard enteral diets (normocaloric or hypercaloric, normoproteic or hyperproteic) and specific enteral diets were used and were chosen according to the clinical and nutritional status of the patient. The diets were free of lactose, sucrose and gluten and were supplemented with a protein supplement when necessary to reach the protein target amount.

To calculate the protein target estimate, the clinical condition of the patient and the recommendation of the American Society for Parenteral and Enteral Nutrition (ASPEN)^(11)^ of 1.2 to 2.0g/kg/day for critically ill adult patients were considered. The ideal target in the unit was a prescription of 1.5g/kg/day; for critically ill obese adults (BMI 30 to 40kg/m^2)^, the ideal target was 2.0g/kg/day; and for patients with BMI > 40kg/m^2^, the ideal target was 2.5g/kg/day.

Enteral nutrition therapy adequacy was assessed by daily recording of the volume of the prescribed enteral formula (planned volume according to the daily prescription by the dietitian, according to the needs calculated for each patient) and the volume administered (actual infused total daily volume), according to the records of the nursing and nutrition teams.

The following calculations were performed to determine the adequacy of the prescribed and infused volumes as well as the percentage of protein adequacy for each patient using ENT.

**Table:** 

adequacy of volume infused (%) = infused volume × 100/prescribed volume
protein adequacy (%) = protein intake × 100/prescribed protein

Protein intake and infused enteral nutrition volume ≥ 80% of the total planned dietary intake were considered adequate.^(12)^

The diagnosis during hospitalization, the reasons for discontinuation of ENT, the time to reach the protein goal and the clinical outcome of the patient (discharge, death or ICU transfer) were also evaluated, and the data were collected from the medical records or from the nursing and nutrition teams' records.

The data were transferred to Microsoft Excel^®^ spreadsheets for Windows version 2013. Statistical analyses were performed using the Statistical Package for Social Sciences (SPSS) software, version 13.0. Continuous variables were tested for normality using the Kolmogorov-Smirnov test. The chi-square test and Fisher's exact test were used to analyze categorical variables. For continuous variables, the paired Student's t-test and the Wilcoxon test were used to compare the means of normally and non-normally distributed dependent groups, respectively. Statistical significance was established at p < 0.05 in all analyses.

This study was guided by the ethical standards for research involving human subjects contained in Resolution 466/2012 of the National Health Council and according to the Declaration of Helsinki of 1975, revised in 2000. It was approved by the Ethics Committee of the *Universidade Federal do Vale do São Francisco* (UNIVASF) under CAAE: 72192917.3.0000.5196.

## RESULTS

The sample consisted of 188 critical patients hospitalized in the ICU with exclusive ENT for at least 72 hours. The sample mainly comprised adult patients (68.1%), with a median age of 48.5 years (31.0 - 63.75 years; minimum 18 and maximum 92 years), and 71.3% of the sample was male. Hospitalization due to trauma (46.3%) and neurological disease (33%) were the most prevalent diagnoses, and hospital discharge (73.4%) was the clinical outcome most recorded in the medical records (Table 1).

**Table 1:** Demographic and clinical characteristics of adult critically ill patients receiving only enteral nutrition therapy

Variables	Total patients n = 188
Age (years)	48.5 (31.0 - 63.75)
Sex	
Male	134 (71.3)
Female	54 (28.7)
Diagnosis at hospitalization	
Trauma	87 (46.3)
Neurological	62 (33.0)
Surgical	11 (5.9)
Sepsis	6 (3.2)
Vascular	10 (5.3)
Cardiological	1 (0.5)
Other	11 (5.9)
Clinical outcome	
ICU discharge	138 (73.4)
Death	47 (25.0)
ICU transfer	3 (1.6)

ICU - intensive care unit. Data are expressed as the median and interquartile range or n (%).

The assessment of nutritional status according to BMI showed that most patients were of normal weight (54.8%); 11.7% were malnourished. The nutritional diagnosis obtained by AC identified a lower prevalence of eutrophic patients (46.4%) and a higher number of malnourished patients (44%), as described in table 2.

**Table 2:** Nutritional parameters of adult critically ill patients receiving only enteral nutrition therapy

Variables	n (%)
Nutritional status (BMI)	
Malnutrition	22 (11.7)
Eutrophy	103 (54.8)
Excess weight	63 (33.5)
Nutritional status (AC)	
Malnutrition	73 (44.0)
Eutrophy	77 (46.4)
Excess weight	16 (9.6)
Adequacy of infused volume	
≥ 80%	173 (92.0)
< 80%	15 (8.0)
Time to reach the protein target	
> 3 days	56 (29.8)
≤ 3 days	132 (70.2)
Protein adequacy	
≥ 80%	82 (43.6)
< 80%	106 (56.4)
Mean administered protein (g/kg/day)	
≥ 1.2g/kg/day	88 (46.8)
< 1.2g/kg/day	100 (53.2)

BMI - body mass index; AC - arm circumference.

The percentage of mean infused enteral diet volume compared to the volume that was prescribed was 90.6 ± 7.4%, and protein adequacy was 72.2 ± 18.2% on average. The time required to achieve the prescribed protein target was 3.21 ± 1.9 days.

Table 2 shows that 92% of patients under ENT received the proposed target (≥ 80%) for the prescribed volume; however, when protein adequacy was analyzed, it was insufficient (< 80%) in 56.4% of cases. Among the critical patients, 70.2% achieved the proposed protein target (1.2 - 2.0g/kg/day) according to the estimated nutritional need in up to three days, but only 46.8% consumed at least 1.2g/kg/day of protein during the ICU stay.

**Figure 1:**
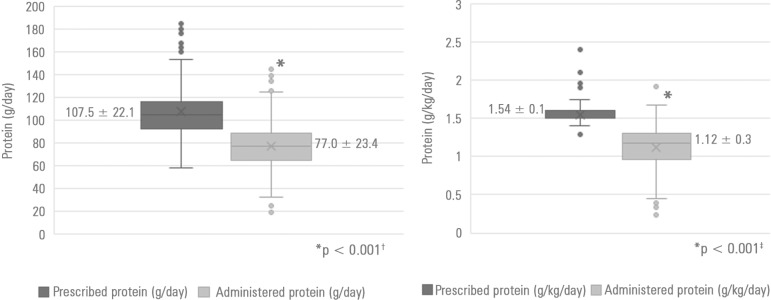
Differences between means of prescribed *versus* administered protein in adult critically ill patients receiving only nutritional therapy. ^†^ Paired Student's test; ^‡^ Wilcoxon test.

Figure 1 shows that the mean protein administered was 77.0 ± 23.4g/day and 1.12g/kg/day, while the prescribed mean protein was 107.5 ± 22.1g/day and 1.54g/kg/day. The differences between the prescribed mean (g/day and g/kg/day) and the administered mean were statistically significant (p < 0.001).

The main reasons described in the medical records for discontinuation of ENT were nausea, vomiting and diarrhea (33.3%), clinical complications (26.7%), fasting for diagnostic or surgical procedures (20.0%), loss of enteral access (6.7%) and others (13.3%).

When evaluating the association of ICU mortality with nutritional parameters (Table 3), we observed that the nutritional status diagnosed by BMI (p = 0.023) and AC (p = 0.041), as well as the protein adequacy rate (p = 0.012), were significantly associated with the clinical outcomes of critically ill patients.

**Table 3:** Mortality occurrence in the intensive care unit and its association with nutritional parameters of adult critically ill patients receiving only enteral nutrition therapy

Variables	Clinical outcome		p value
	Death	Nondeath	
Nutritional status (BMI)			
Malnutrition	8 (16)	14 (10)	0.023*
Eutrophy	33 (66)	70 (51)	
Excess weight	9 (18)	54 (39)	
Nutritional status (AC)			
Malnutrition	26 (60)	47 (38)	0.041*
Eutrophy	14 (33)	63 (51)	
Excess weight	3 (7)	13 (11)	
Adequacy of infused volume			
≥ 80%	44 (88)	129 (93.5)	0.232†
< 80%	6 (12)	9 (6.5)	
Time to reach the protein target			
> 3 days	13 (26)	43 (31)	0.589†
≤ 3 days	37 (74)	95 (69)	
Protein adequacy			
≥ 80%	14 (28)	68 (49)	0.012†
< 80%	36 (72)	70 (51)	
Mean administered protein (g/kg/day)			
≥ 1.2g/kg/day	3 (6)	7 (5)	0.727†
< 1.2g/kg/day	47 (94)	131 (95)	

BMI - body mass index; AC - arm circumference.

* Chi-square test;

† Fisher's exact test. Results expressed as n (%).

## DISCUSSION

The evaluated population comprised mostly adult male patients who suffered trauma secondary to external causes and remained in the ICU for physical and mental rehabilitation. The high prevalence rates found corroborate the data reported in the literature, and the main group of causes of hospital admissions for the male population was external causes characterized by injuries resulting from traffic-related accidents, drowning, poisoning, falls or burns and violence.^(13)^

In the present study, nutritional assessment was performed using objective methods (BMI and AC) and showed different rates of malnutrition in the ICU. The prevalence found by AC was approximately four times higher than that obtained by BMI. This difference between the methods was also found by Martins et al.,^(14)^ who observed that AC diagnosed twice as much malnutrition compared to BMI.

These results demonstrate greater sensitivity of AC in identifying malnutrition in critically ill patients and reinforce the importance of using several methods to define a more precise nutritional diagnosis. There are many limitations when using a specific method in patients admitted to the ICU, such as changes in body fluids and difficulties in measuring and obtaining reliable data. Despite the relevance of nutritional assessment in critically ill patients, there is still no gold standard in the literature for nutritional assessment of these patients.^(15)^

The evaluation of the prescribed *versus* infused volume revealed that the majority of patients received the proposed target (≥ 80%) and that the mean infused enteral nutrition (90.6%) was higher than the values reported by Santos et al. (82.9%),^(16)^ Ribeiro et al. (81.6%),^(17)^ and Stefanello & Poll (78.0%).^(18)^

The mean time to reach the estimated protein target in this study was lower than the mean time of 3.75 ± 2.25 days recorded by Santana et al.^(1)^ and higher than that found by Lins et al.^(19)^ (2.51 ± 2.92 days). When analyzing the group of individuals who received the estimated protein recommendations within three days, the percentage (70.2%) was higher than that described by Pasinato et al. (2011).^(20)^

Achieving nutritional targets early is one of the recommendations in international guidelines for critical patients using ENT, especially in patients with high nutritional risk (Nutritional Risk Screening - NRS, 2002 ≥ 5 or Nutrition Risk in the Critically Ill score - NUTRIC ≥ 5) or who are malnourished.^(11)^ Similarly, the nutritional recommendations for patients who have suffered brain trauma should also be reached early, preferably between the fifth and seventh days postinjury, as studies have shown a significant association with mortality reduction in these patients.^(21)^

The percentage of mean protein adequacy in this study was lower than that of other ICU studies performed in Brazil.^(17,19,22,23)^ The results regarding inadequate protein intake demonstrate the difficulty of achieving the proposed nutritional targets, which may lead to increased hospital malnutrition, increased complications and worsening of the clinical outcome, which was also demonstrated in this study.^(1,5)^

Current recommendations suggest that critically ill patients hospitalized in ICUs should receive hyperproteic diets containing a protein content of at least 1.2g/kg/day;^(11)^ however, achieving this recommendation is a major challenge. In this study, more than half of the patients were unable to receive the minimum recommended protein intake for varying reasons. Enteral formulas frequently need to be supplemented with protein because few enteral formulas have a satisfactory protein supply and meet the nutritional recommendations without exceeding the caloric requirement (30kcal/kg/day).^(11)^ In addition, enteral diets are administered intermittently with a nocturnal pause. It is impossible to compensate for the daily interruptions during the night, which makes it difficult to comply with the proposed nutritional target.

The inadequacy of ENT needs to be monitored by quality clinical indicators to identify problems and promote the improvement of care provided, including quality and patient safety. The individual monitoring of enteral nutrition interruption factors by a multidisciplinary team would improve the nutrition offered to these patients and consequently improve quality of life and reduce malnutrition, length of hospitalization and hospital costs.^(24)^

Among the professionals involved in the success of ENT, the role of the nursing team (nurses and technicians) in the control and monitoring of adverse factors that prevent the administration of the prescribed enteral diet should be emphasized. In this study, gastrointestinal complications were the main causes of interruption of ENT, followed by clinical complications and fasting for procedures, which did not corroborate the findings of Santana et al.^(1)^ and Rocha et al.,^(25)^ who identified fasting for procedures as the main cause of interruption. Thus, the need for a trained and involved nursing team is emphasized because the active participation of nurses in the formulation of nutritional plans for critically ill patients plays an important role in achieving the goals of nutritional therapy.^(26)^

The association of nutritional status with the clinical outcome of patients in a hospital environment has been described previously in the literature, and this scientific evidence supports the need for constant monitoring. Malnutrition significantly increases hospital mortality in both critically ill and noncritically ill patients, and an accurate nutritional assessment is essential to optimize clinical outcomes. For this reason, an early nutritional assessment protocol should be included at the time of admission to the hospital as part of the clinical treatment of these patients.^(27)^

The efficacy of nutritional therapy depends on adjusting the calorie and protein intake to the actual condition of the patient, monitoring the adequacy of nutritional support and minimizing the risk of mortality and morbidity resulting from malnutrition.^(15)^ The results of this study showed relevant associations of nutritional status and protein intake with the mortality of critically ill patients. These results corroborate previously reported data showing that the use of higher protein concentrations (> 1.2g/kg/day) is associated with reduced morbidity and mortality in this population.^(14,28,29)^

There is evidence that adequate protein intake is more important than calorie intake for critically ill patients, and meeting the protein target should be considered a priority to support the metabolic demands of organ function, wound healing and immunological function.^(30)^ Protein intake of less than 0.8g/kg/day during ICU and hospital stays is associated with worse outcomes and higher mortality rates over a period of six months.^(31,32)^

It is clear that nutritional support should be considered adjuvant therapy in the treatment of critically ill patients, as it has a positive impact on reducing hospital malnutrition and can significantly affect the clinical outcomes of these patients. Thus, achieving nutritional goals during the ICU stay should be a priority in the treatment of critically ill patients.

## CONCLUSION

Early nutritional status assessment allows the identification of critically ill patients who need more aggressive nutritional intervention, and this study again demonstrates the importance of nutritional diagnosis for these patients, given the significant association of this parameter with clinical outcomes in the intensive care unit. Adequate protein intake may influence the outcomes of these patients, but despite the efforts of multidisciplinary teams, critically ill patients often do not receive the prescribed enteral nutrition, especially with respect to protein content. This deficiency seems to interfere with the prognosis of these patients, demonstrating how this practice may be harmful to critically ill patients receiving enteral nutrition therapy.
